# Test–Retest Reliability of an iPhone^®^ Inclinometer Application to Assess the Lumbar Joint Repositioning Error in Non-Specific Chronic Low Back Pain

**DOI:** 10.3390/ijerph18052489

**Published:** 2021-03-03

**Authors:** Alejandro Caña-Pino, Luís Espejo-Antúnez, José Carmelo Adsuar, María Dolores Apolo-Arenas

**Affiliations:** 1 Department of Medical Surgical-Therapy, Medicine Faculty, Extremadura University, 06006 Badajoz, Spain; alejandrocp@unex.es (A.C.-P.); luisea@unex.es (L.E.-A.); mdapolo@unex.es (M.D.A.-A.); 2 Promoting a Healthy Society Research Group, Faculty of Sport Sciences, University of Extremadura, 10003 Cáceres, Spain

**Keywords:** non-specific low back pain, proprioception, joint position sense, reliability, smart phone

## Abstract

**Background:** The joint position sense (JPS) has been used as an indirect marker of proprioception in subjects with non-specific chronic low back pain (NSCLBP), showing impairment in previous studies. It seems necessary to devise reliable tests to measure proprioceptive deficits in subjects with NSLBP. The objective of this study was to analyse the test–retest reliability and smallest real difference (SRD) of lumbar proprioception through the JPS indicator in a sample of patients with NSCLBP. **Methods:** Fifty participants with NSCLBP performed three repetitions of 30° lumbar flexion while standing and sitting using the iPhone^®^ inclinometer application to measure the lumbar joint repositioning error. For the reliability analysis, we performed an intra-session test–retest. **Results**: The total sample ICC values were excellent for standing (0.96) and sitting (0.93) 30° lumbar flexion. In addition, our results showed that, for the total sample, an SRD < 12% can be considered as a true change in proprioception concerning this procedure. On the other hand, men have better reliability than women in both standing and sitting positions. Additionally, the sitting position has better reliability than the standing position. The standard error of measurement (SEM) percentage was 4.2 for standing and 3.8 for sitting. The SRD percentage was 11.6 for standing and 10.4 for sitting. **Conclusions:** The iPhone^®^ inclinometer seems reliable for assessing proprioceptive ability through the lumbar joint repositioning error in subjects with NSCLBP in both standing (ICC = 0.96) and sitting (ICC = 0.93) positions. This technological device showed a lower measurement error for sitting position (SRD < 12%).

## 1. Introduction

Currently, numerous studies address non-specific chronic low back pain (NSCLBP), however, the battery of tests and measuring instruments used for the evaluation of physical and psychological fitness is varied. NSCLBP is defined as persistent back and sacrum pain lasting more than 12 weeks; it occurs in 85% of cases for unknown reasons, is unrecognizable, and affects 12–33% of the adult population [1].

Although the most studied etiologic factors in NSCLBP include psychosocial factors [2], lack of self-efficacy [3], and changes in postural and proprioceptive control [3,4,5,6,7,8], the large number of external indicators or variables (i.e., variability in the recording and measurement instrument used) as well as internal factors (i.e., age, stage of development or professional work performed) have been shown to influence parameters related to postural control [7,8], making it difficult to reach a consensus on which instruments and parameters to use in the practical management of pathology. Postural control has been defined as the ability to maintain, obtain or re-establish balance in any posture or activity through information processing by the visual, vestibular, and proprioceptive system [9]. Postural control deficits have been detected in subjects with NSCLBP during the first three months of evolution, remaining even after the symptoms have subsided [4,7,8]. 

Individuals with NSCLBP have shown structural (i.e., muscle atrophy and fat infiltration) and functional (i.e., altered neuromuscular coordination between the superficial and deep back muscles) changes in the trunk muscles [10,11]. These changes lead to alterations in proprioception, deficits in tactile discriminatory acuity leading to interference with motor control [4,12,13]. According to Romero-Franco et al., optimal motor control requires excellent proprioceptive skills to recognize and reproduce corporal positions [14]. Proprioception is defined as “the perception of joint and body movement as well as position of the body, or body segments, in space” [15]. This definition differs from the concept of kinaesthetic sense by referring specifically to “the sense of position and movement of our limbs” [16]. Proprioception is an essential aspect of balance. Impaired proprioception has been shown to affect normal coordinated movement; there may be an increased risk of injury as the intensity of low back pain and disability increases [17]. Several studies have shown alterations in the proprioception of the lumbopelvic region in patients with NSCLBP [5,6,18,19]. In this sense, proprioceptive decline probably leads to an increased reliance on the visual and vestibular system during a static bipedal stance [5,19,20,21,22].

When applying for therapeutic exercise programs in patients with NSCLBP, the only way to affirm if back muscle and lumbar proprioception is being improved is to assess local proprioceptive errors with specific tests such as joint position sense (JPS) [23,24,25]. The JPS test measures how well a participant can replicate a “target position” of the lumbar spine. These are presented through visual feedback, manual guidance, or verbal feedback. After presentation of the target position, the participant is moved out of the position and asked to replicate it actively [26]. In addition to the interest in proprioceptive evaluation, there is no consensus as to the most appropriate posture and measurement instrument in subjects with NSCLBP [26,27,28]. In this sense, to date, there is insufficient evidence to associate a specific position (e.g., sitting or standing) to the suffering of low back pain [29,30]. Equally, no user-friendly technological device is available either, which would be reliable and valid for assessing proprioceptive error in this type of subject [26,28].

In recent years, the knowledge of different modalities of therapeutic exercise (discriminative perceptual exercise, sensorimotor exercises with the tactile stimulus, dissociative exercises, proprioceptive exercises, etc.) has increased significantly; however, the outcome measures analysed were pain, disability, quality of life, and return to work [31]. Studies measuring the degree of proprioceptive acuity are limited to improvement in motor control [32]. Among these is the degree in joint repositioning error as the most widely used outcome measure; however, to date, there is no gold standard procedure for its measurement. This could be the reason why there is no clear evidence of the impact of therapeutic interventions on proprioceptive deficits in subjects with NSLBP [13,33]. 

In light of this, it seems necessary to devise reliable tests to measure proprioceptive deficits in subjects with NSCLBP.

The JPS has been used as an indirect marker of proprioception in subjects with NSCLBP, showing impairment in previous studies [23,24,25]. Since this test evaluates the capability to recognize and reproduce lumbar spine positions, it often requires the time-consuming task of analysing images through specific software and/or sophisticated instruments such as an isokinetic dynamometer, inertial sensors, an electrogoniometer, or photo-analysis (high-resolution camera) to monitor proprioceptive deficits in people with NSCLBP [14,34]. Although these types of measuring equipment are reliable and valid, they are not portable and their installation is time-consuming [35]. For this reason, some authors have designed and validated methods to quickly and easily obtain proprioceptive errors by developing smartphone applications based on the inclinometer integrated into smartphones [14,35]. For measurement, the iPhone^®^ has been used as an inclinometer in other populations and their reliability has been evaluated for mobility on people with cervical spine [36,37], lumbar spine [38,39,40], and thoracic spine [41] pathologies; standing lumbar lordosis [42]; ankle dorsiflexion [43,44] and inter-limb asymmetries [44]; active wrist range of motion in asymptomatic subjects [45]. To date, there is no gold standard instrument for the assessment of JPS in the lumbar region of subjects with NSCLBP.

To the best of our knowledge, no previous studies have evaluated the reliability, smallest real difference (SRD), and standard measurement error (SEM) concerning lumbar proprioception in patients with NSCLBP measured with the iPhone^®^ inclinometer application. Therefore, the objective of this study was to analyse the test–retest reliability and SRD of lumbar proprioception through the JPS indicator in a sample of patients with NSCLBP, by calculating the intraclass correlation coefficient (ICC) for relative reliability and SRD and SEM for absolute reliability.

## 2. Materials and Methods 

### 2.1. Sample Size 

A sample size of 37 participants with two observations per participant achieves 95% power to detect an intra-class correlation of 0.91 (excellent reliability) under the alternative hypothesis when the intra-class correlation under the null hypothesis is 0.75 (good reliability) using an F-test with a significance level of 0.05. The null and alternative hypotheses were established following the study by Walter et al. [46]. 

### 2.2. Participants

A total of 50 participants (25 females and 25 males) were included in the study after verifying that they met the inclusion criteria. Inclusion criteria included: (a) being aged between 18 and 45 years [47], (b) experiencing NSCLBP for ≥3 months [1,48], and (c) speaking Spanish as their native language. The diagnosis of NSCLBP was made by a physician. Participants suffering from “pain between the costal margins and the inferior gluteal folds with or without referred pain to the leg [49] were included, provided they scored at least 3/10 on the numerical pain rating scale (NPRS) (ranged from 0–10, with 0 representing no pain and 10 representing the worst pain) [50]. Patients with NSCLBP were allowed to have referred pain in the leg above the knee as long as no neurological symptoms were present [51]. 

The exclusion criteria included [1]: (a) pregnancy, including six months postpartum; (b) a history of back or lower limb surgery; (c) signs of symptoms of neuropathic pain (e.g., a painful radiculopathy) [51]; (d) trauma to the back or lower extremities in the last three months; (e) metal spine implants; (f) neurological or vestibular disorders; (g) consumption of drugs with a potential effect on balance in the 24 h before the study; (h) a diagnosed psychiatric disorder or severe cognitive impairment. 

The study was approved by the ethics committee of the University of Extremadura, reference number 77//2018. The study was performed following the updates to the Helsinki Declaration, modified by the 64th General Assembly of the World Medical Association (Fortaleza, Brazil, 2013). 

### 2.3. Measurements 

Several measurements were collected for sample characterization. First, we asked about age, time since the onset of low back pain, and its intensity through the numerical pain rating scale (NPRS). Second, the participant’s bodyweight (kg) and height (cm) were measured without shoes. Body mass index (BMI) was calculated according to the following formula: BMI = weight (Kg)/height^2^ (m). JPS (degrees) was used to assess the lumbar joint repositioning error (The JPS is the measurement described in the Reliability Procedures section). 

The iPhone^®^ 10 (Apple Inc, Cupertino, CA, USA) was used as a measuring instrument with level application (iHandy© level). The iHandy^®^ level application is integrated into the iPhone^®^ and is a free application with a visual display similar to that of a digital inclinometer in regard to numeric size. The application uses the iPhone^®^‘s built-in accelerometer and a digital display to display the angle measured. There is no reported accuracy of this application by the manufacturer. The unit of measurement was degrees (°). 

### 2.4. Reliability Procedures

**Figure 1:**
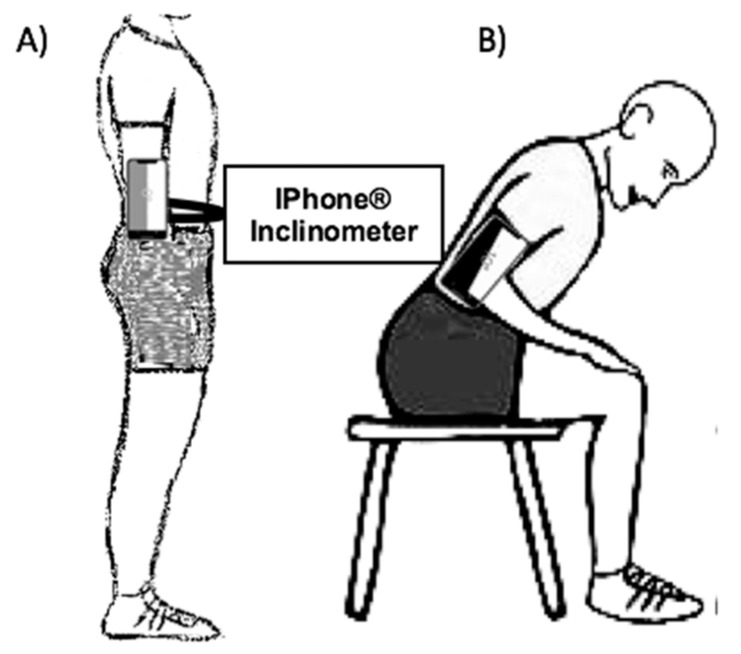
Evaluation with iPhone^®^ inclinometer. (**A**) Initial position: standing 0° lumbar flexion; (**B**) Final position: sitting 30° lumbar flexion.

The iPhone^®^ inclinometer parameters, as well as the participant’s position, to assess the lumbar joint repositioning error through the JPS, were set according to the following instructions (Figure 1): (i) The standing position was with the arms alongside the body, their feet externally rotated in relation to the progression line (approximately 20 degrees), their heels three centimetres apart, to look at a fixed point at eye level. (ii) The seated position was in a height-adjustable seat according to the length of the shank, without a backrest, feet resting on the ground and arms resting on the front of the thighs. The feet were kept with the same distance/rotation as in the standing condition. (iii) The iPhone^®^ was placed in an upright position immediately above the iliac crest, at a point midway between the anterior superior and posterosuperior iliac spine, fixed with a belt; (iv) the inclination of the inclinometer was 0°; (v) the 0° position of the inclinometer was set as the starting position. (vi) The range of movement was 0 to 30°, where the subject was passively led by the evaluator to a 30° flexion; (vii) the subject was to memorize this position for 10 s; (viii) the subject actively returned to the initial position. (ix) Later, the subject had to actively reproduce the position in three repetitions. The first repetition was a practice trial for familiarisation with the test and was therefore a non-valid trial. In the reliability analyses, the second trial was used as a “test” and the third trial was used as a “retest”. The time between attempts was five minutes. The order of the standing and seated evaluations was randomly determined. The time between evaluations was 10 min. The evaluations were conducted in an environment without visual or auditory signals. All evaluations were performed by a single evaluator with a graduate degree in physical therapy and previous experience. 

### 2.5. Statistical Analysis

The variables included in the study followed a normal distribution. The Shapiro–Wilks test was carried out to check the distribution of data. Data concerning the characterization of the sample were given as the mean and standard deviation. Student’s t-test was used to see if there were statistically significant differences between test and retest, and also between genders. The significance level was determined at *p* < 0.05.

The reliability was studied through relative reliability and absolute reliability statistics.

The relative reliability was determined by the intraclass correlation coefficient (ICC)_3,1_ [31]. The ICC data were calculated using the next parameters: (i) model: two-way random effects; (ii) type: single rater and, (iii) definition: consistency [52]. 

The following classification was used for interpreting the ICC [53]: an ICC less than 0.5 corresponds to poor reliability, an ICC from 0.5 to 0.75 corresponds to moderate reliability, an ICC from 0.75 to 0.9 corresponds to good reliability, and an ICC greater than 0.9 corresponds to excellent reliability.

Absolute reliability was determined by SEM and SRD. No change below SEM can be considered real [54]. The SEM was calculated with the following formula: SEM = SD·$\sqrt{1-ICC}$ where SD is the mean SD of the two repetitions. The SEM% was calculated with the formula: SEM% = SD√1 − ICC/Mean_test1&test2_ × 100 [55]. The SRD formula was SRD = 1.96·SEM·$\sqrt{2}$. This score was subsequently turned into a percentage with the formula: SRD% = 1.96·SEM·$\sqrt{2}$/Mean_test1&test2_ × 100 [55]. The SEM% and SRD% allows comparison of SEM and SRD with other instruments to measure the proprioceptive deficits [55]. 

Bland–Altman analyses were performed to show the level of agreement between tests and retests regarding standing and sitting lumbar flexion. In these graphics, the *x*-axis represents the mean of the test and the *y*-axis shows the difference between the two measurements (1–2; 1 = test; 2 = retest). Plots show “the bias” and limits of agreement (LOA) calculated to 95%. Bias values close to zero represent strong agreement and the smaller range between these two LOA are interpreted as better agreement [56].

## 3. Results

Table 1 includes the characteristics of the participants for the total sample and men and women sub-groups.

Table 2 shows the descriptive data of the test and retest measures. No significant differences were observed between the test and retest measures of either standing or sitting. There were also no significant differences for the men and women subgroups in any of the variables studied.

Table 3 shows relative reliability (ICC) and absolute reliability (SEM, SEM%, SRD, and SRD%). Total sample ICC values were excellent for standing (0.96) and for sitting (0.93) 30° lumbar flexion. The ICC was slightly better for standing than for sitting.

SEM% was 4.2 for standing and 3.8 for sitting. On the other hand, SRD% was 11.6 for standing and 10.4 for sitting. 

In the men sub-group, ICC values were excellent (>0.96) for two variables, both standing and sitting lumbar flexion. SEM% were around 1.5% for standing and 0.75% for sitting. SRD% were around 12% for standing and 7% for sitting. 

In the women sub-group, ICC values were excellent (>0.95) for standing and good (>0.80) for sitting lumbar flexion. SEM% were around 2% for standing and 1% for sitting. SRD% were around 18% for standing and 13% for sitting.

The ICC was slightly better for standing than sitting lumbar flexion. 

The results obtained show that the lumbar joint repositioning error appears to be sensitive to changes in position, showing a detection in the lower measurement error for the sitting position (SRD < 12%).

**Figure 2:**
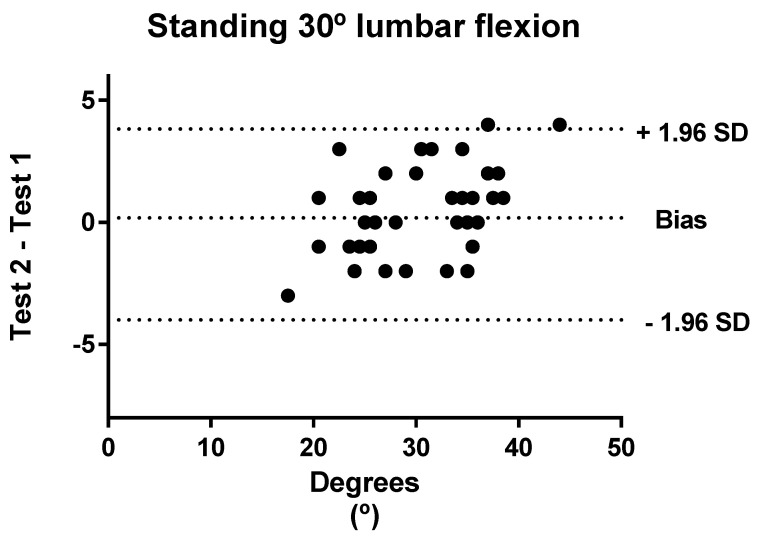
Bland–Altman plots with the bias and the limits of agreement for standing 30° lumbar flexion.

Figure 2 shows the Bland–Altman plots of the standing 30° lumbar flexion. The Bland–Altman plots indicated that the points outside the 95% LOA were less than 2%. 

**Figure 3:**
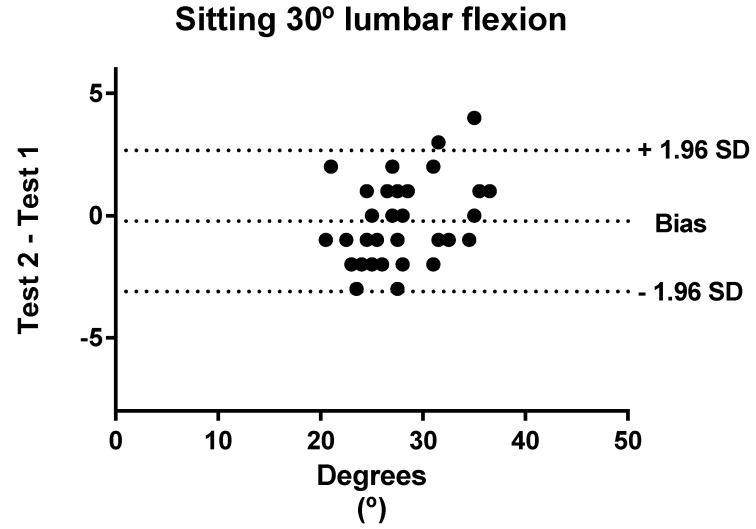
Bland–Altman plots with the bias and the limits of agreement for sitting 30° lumbar flexion.

Figure 3 shows the Bland–Altman plots for sitting 30° lumbar flexion. The Bland–Altman plots indicated that the points outside the 95% LOA were less than 4%.

## 4. Discussion

According to our best knowledge, the reliability of two trials (test/retest) of standing and sitting 30° lumbar flexion has not been previously reported regarding NSCLBP measured with the iPhone^®^ inclinometer. The total sample ICC values were excellent for both standing (0.96) and sitting (0.93) 30° lumbar flexion. In addition, our results showed that, for the total sample, an SRD < 12% can be considered as a true change in proprioception concerning this procedure. On the other hand, men have better reliability than women in both standing and sitting positions. Also, the sitting position has better reliability than the standing position. 

The absolute reliability data (SEM and SRD) obtained in this study are excellent. These results could perhaps help to improve knowledge about which changes could be considered beneficial after therapeutic intervention, or, on the contrary, to consider these changes as normal measurement variability. From a clinical point of view, previous studies such as that of Georgy et al. [34] and Kolber et al. [38] measured this same variable in subjects with NSCLBP and healthy subjects. They obtained absolute error values lower than those shown in this study. Therefore, it is not possible to affirm real changes in proprioception. The results obtained in the present study in terms of SRD could allow the clinical interpretation of other studies. Other studies, such as that of Kolber et al., did not show statistically significant differences for the JPS after performing a comparison between the iPhone^®^ inclinometer and gravity-based bubble inclinometer in healthy subjects. They reported high concurrent validity, (ICC = 0.86). In our study, we obtained an excellent ICC for standing (ICC = 0.96) and sitting (ICC = 0.93) with 95% confidence. This could be explained by the fact that the changes detected are lower than the SRD%, obtained in the present study (standing: 4.2; sitting: 3.8). On the other hand, Georgy et al. observed no differences for this variable measured with an isokinetic dynamometer between healthy subjects and subjects with NSCLBP, disagreeing with the conclusions reached by these authors. These results appear consistent with recent systematic reviews and meta-analyses that state the lack of conclusive results with respect to this variable in subjects with NSCLBP [26,28]. 

Currently, there are different methods for measuring lumbar proprioception. In addition to the JPS test as the most commonly used procedure, there is also the threshold to detection of passive motion (TDPM) and directional motion perception (DMP). The TDPM is a proprioception procedure that measures sensitivity to the detection of movement. Starting from a neutral lumbar spine posture, participants undergo passive lumbar movement in custom devices at a constant velocity and indicate the earliest point that they sense a positional change. This can be combined with DMP, where participants indicate the direction of the passive movement. The outcome measures are the smallest range of motion (ROM) at which the participant reported movement (TDPM) and the direction of movement reported compared with the correct direction (DMP) [26]. 

Regarding the measuring instrument, some studies report non-statistically significant results when comparing a healthy subject to NSCLBP, concluding that the measurement instrument was not sufficiently accurate and there was heterogeneity in the sample [26,28,57]. In our study, the inclinometer uses a fixed vertical reference point made by gravity, so it is stable as long as the zero points are accurately calibrated and set [38]. With the rise of smartphone ownership among therapists, there is a greater opportunity to use devices to support clinical decision-making [58]. Several studies have examined the validity and reliability of goniometer applications based on smartphones designed to measure the ROM, taking into account the three most common factors affecting the measurement: the patient, the device, and the examiner [36,37,39,42,44,45,58].

On the other hand, taking into account the valuation position, the results observed for the sitting position show somewhat higher reliability values than the standing position. This position has been associated with the presence of chronic low back pain in sedentary workers [27]. Although there is no clear evidence that a specific posture is the cause of low back pain, there is no clear evidence that a specific posture causes low back pain [59]; these results could be used to assess lumbar repositioning errors in working subjects that are seated for the majority of their working day. Future studies are needed to determine this aspect.

Some potential limitations need to be addressed. As for external validity, it is not possible to generalize the data to another type of population with low back pain because the functional capacity of these patients depends on other factors, such as psychosocial factors. The absence of an inter-evaluator measurement and the measurement on different days so that the patient can become familiar with the protocol is recommended.

## 5. Conclusions

The iPhone^®^ inclinometer seems reliable for assessing proprioceptive ability through the lumbar joint repositioning error in subjects with NSCLBP in both standing (ICC = 0.96) and sitting (ICC = 0.93) positions. This technological device showed a lower measurement error for the sitting position (SRD < 12%).

## Figures and Tables

**Table 1:** Characteristics of the study participants.

	Total (*n* = 50)	Men (*n* = 25)	Women (*n* = 25)	p *
Age (years)	33.58 (7.29)	32.16 (7.53)	35 (6.88)	0.170
Height (cm)	174.30 (0.14)	174.84 (0.13)	172.64 (0.14)	0.225
Weight (kg)	71.40 (5.20)	71.52 (5.20)	70.48 (5.26)	0.486
BMI (kg/m^2^)	24.49 (3.09)	24.05 (2.18)	24.93 (3.79)	0.321
Intensity of pain perception (NPRS)	6.9 (1.2)	7.3 (1.0)	6.6 (1.4)	0.068
Average pain duration (months)	37.5 (36.7)	28.1 (28.4)	46.8 (41.9)	0.071
Standing joint position sense (°)	5.55 (3.07)	5.57 (2.91)	5.52 (3.28)	0.953
Sitting joint position sense (°)	4.22 (2.52)	4.19 (2.47)	4.25 (2.63)	0.927

* Student’s *t*-test; *p* < 0.05: statistical significance; cm: centimetres; kg: kilograms; m: metres; NPRS: The scale ranges from 0 (“no pain at all”) to 10 (“worst imaginable pain”); °: degrees. Table 1 includes age, anthropometric measurements, body composition, intensity of pain perception, average pain duration, and joint position sense.

**Table 2:** Summary of iPhone^®^ Inclinometer.

iPhone^®^ Inclinometer			
	Measure 1	Measure 2	
Test Measurement	Mean (SD)	Mean (SD)	p *
**All participants**			
Standing 30° lumbar flexion (°)	30.14 (5.87)	30.44 (6.81)	0.244
Sitting 30° lumbar flexion (°)	27.84 (3.67)	27.62 (4.23)	0.297
**Men**			
Standing 30° lumbar flexion (°)	30.80 (6.53)	31.16 (7.82)	0.376
Sitting 30° lumbar flexion (°)	28.68 (4.22)	28.52 (4.48)	0.476
**Women**			
Standing 30° lumbar flexion (°)	29.48 (5.18)	29.72 (5.68)	0.465
Sitting 30° lumbar flexion (°)	27 (2.86)	26.72 (3.84)	0.442

* Student’s *t*-test.; *p* < 0.05: statistical significance; °: degrees; SD: Standard Deviation.

**Table 3:** Test–Retest Reliability of standing and sitting lumbar flexion in 2 measurements with an interval of minutes between measurements.

All (*n* = 50)	iPhone^®^ Inclinometer				
Assessed Action	ICC (95% CI)	SEM (°)	SEM (%)	SRD (°)	SRD (%)
Standing 30° lumbar flexion	0.96 (0.93–0.98)	1.27	4.2	3.51	11.6
Sitting 30° lumbar flexion	0.93 (0.88–0.96)	1.05	3.8	2.90	10.4
Men (*n* = 25)					
Standing 30° lumbar flexion	0.96 (0.92–0.98)	1.40	4.5	3.88	12.5
Sitting 30° lumbar flexion	0.97 (0.93–0.99)	0.78	2.7	2.16	7.5
Women (*n* = 25)					
Standing 30° lumbar flexion	0.96 (0.91–0.98)	1.90	6.4	5.26	17.8
Sitting 30° lumbar flexion	0.86 (0.71–0.94)	1.24	4.6	3.45	12.8

ICC: Intra-class Correlation Coefficient; CI: Confidence Interval; SEM: Standard Error Measurement SRD: Small Real Difference; °: degrees; %: percentage.

## Data Availability

Not applicable.

## References

[1] Alsufiany M.B., Lohman E.B., Daher N.S., Gang G.R., Shallan A.I., Jaber H.M. (2020). Non-specific chronic low back pain and physical activity: A comparison of postural control and hip muscle isometric strength. Medicine.

[2] Carragee E.J., Alamin T.F., Miller J.L., Carragee J.M. (2005). Discographic, MRI and psychosocial determinants of low back pain disability and remission: A prospective study in subjects with benign persistent back pain. Spine J..

[3] La Touche R., Grande-Alonso M., Arnes-Prieto P., Paris-Alemany A. (2019). How Does Self-Efficacy Influence Pain Perception, Postural Stability and Range of Motion in Individuals with Chronic Low Back Pain?. Pain Phys..

[4] Van Dieën J.H., Koppes L.L.J., Twisk J.W.R. (2010). Postural sway parameters in seated balancing; their reliability and relationship with balancing performance. Gait Posture.

[5] Brumagne S., Cordo P., Lysens R., Verschueren S., Swinnen S. (2000). The role of paraspinal muscle spindles in lumbosacral position sense in individuals with and without low back pain. Spine.

[6] Descarreaux M., Blouin J.-S., Teasdale N. (2005). Repositioning accuracy and movement parameters in low back pain subjects and healthy control subjects. Eur. Spine J..

[7] Sung W., Abraham M., Plastaras C., Silfies S.P. (2015). Trunk motor control deficits in acute and subacute low back pain are not associated with pain or fear of movement. Spine J..

[8] Da Silva R.A., Vieira E.R., Carvalho C.E., Oliveira M.R., Amorim C.F., Neto E.N. (2016). Age-related differences on low back pain and postural control during one-leg stance: A case-control study. Eur. Spine J..

[9] Caña-Pino A., Apolo-Arenas M.D., Moral-Blanco J., De la Cruz-Sánchez E., Espejo-Antúnez L. (2017). A novel determination of energy expenditure efficiency during a balance task using accelerometers. A pilot study. Assist. Technol..

[10] Caffaro R.R., França F.J.R., Burke T.N., Magalhães M.O., Ramos L.A.V., Marques A.P. (2014). Postural control in individuals with and without non-specific chronic low back pain: A preliminary case-control study. Eur. Spine J..

[11] Hodges P.W., Danneels L. (2019). Changes in Structure and Function of the Back Muscles in Low Back Pain: Different Time Points, Observations, and Mechanisms. J. Orthop. Sports Phys. Ther..

[12] Moseley G.L., Hodges P.W. (2005). Are the changes in postural control associated with low back pain caused by pain interference?. Clin. J. Pain.

[13] Luomajoki H.A., Bonet Beltran M.B., Careddu S., Bauer C.M. (2018). Effectiveness of movement control exercise on patients with non-specific low back pain and movement control impairment: A systematic review and meta-analysis. Musculoskelet. Sci. Pract..

[14] Romero-Franco N., Jiménez-Reyes P., González-Hernández J.M., Fernández-Domínguez J.C. (2020). Assessing the concurrent validity and reliability of an iPhone application for the measurement of range of motion and joint position sense in knee and ankle joints of young adults. Phys. Ther. Sport.

[15] Guerraz M., Provost S., Narison R., Brugnon A., Virolle S., Bresciani J.-P. (2012). Integration of visual and proprioceptive afferents in kinesthesia. Neuroscience.

[16] El-Wishy A., Elsayed E. (2012). Effect of Proprioceptive Training Program on Balance in Patients with Diabetic Neuropathy: A controlled randomized study. Bull. Fac. Phys. Ther..

[17] Zheng Y.-L., Wang X.-F., Chen B.-L., Gu W., Wang X., Xu B., Zhang J., Wu Y., Chen C.-C., Liu X.-C. (2019). Effect of 12-Week Whole-Body Vibration Exercise on Lumbopelvic Proprioception and Pain Control in Young Adults with Nonspecific Low Back Pain. Med. Sci. Monit..

[18] Claeys K., Dankaerts W., Janssens L., Pijnenburg M., Goossens N., Brumagne S. (2015). Young individuals with a more ankle-steered proprioceptive control strategy may develop mild non-specific low back pain. J. Electromyogr. Kinesiol..

[19] Shanbehzadeh S., Salavati M., Talebian S., Khademi-Kalantari K., Tavahomi M. (2018). Attention demands of postural control in non-specific chronic low back pain subjects with low and high pain-related anxiety. Exp. Brain Res..

[20] Mann L., Kleinpaul J.F., Pereira Moro A.R., Mota C.B., Carpes F.P. (2010). Effect of low back pain on postural stability in younger women: Influence of visual deprivation. J. Bodyw. Mov. Ther..

[21] Hamacher D., Hamacher D., Schega L. (2014). A cognitive dual task affects gait variability in patients suffering from chronic low back pain. Exp. Brain Res..

[22] Mok N.W., Brauer S.G., Hodges P.W. (2004). Hip strategy for balance control in quiet standing is reduced in people with low back pain. Spine.

[23] Puntumetakul R., Chalermsan R., Hlaing S.S., Tapanya W., Saiklang P., Boucaut R. (2018). The effect of core stabilization exercise on lumbar joint position sense in patients with subacute non-specific low back pain: A randomized controlled trial. J. Phys. Ther. Sci..

[24] Wang X.-Q., Gu W., Chen B.-L., Wang X., Hu H.-Y., Zheng Y.-L., Zhang J., Zhang H.-Y., Chen P.-J. (2019). Effects of whole-body vibration exercise for non-specific chronic low back pain: An assessor-blind, randomized controlled trial. Clin. Rehabil..

[25] Kong Y.-S., Jang G.-U., Park S. (2015). The effects of prone bridge exercise on the Oswestry disability index and proprioception of patients with chronic low back pain. J. Phys. Ther. Sci..

[26] Tong M.H., Mousavi S.J., Kiers H., Ferreira P., Refshauge K., van Dieën J. (2017). Is There a Relationship Between Lumbar Proprioception and Low Back Pain? A Systematic Review with Meta-Analysis. Arch. Phys. Med. Rehabil..

[27] Bontrup C., Taylor W.R., Fliesser M., Visscher R., Green T., Wippert P.-M., Zemp R. (2019). Low back pain and its relationship with sitting behaviour among sedentary office workers. Appl. Ergon..

[28] Villafañe J.H., Zanetti L., Isgrò M., Cleland J.A., Bertozzi L., Gobbo M., Negrini S. (2015). Methods for the assessment of neuromotor capacity in non-specific low back pain: Validity and applicability in everyday clinical practice. J. Back Musculoskelet. Rehabil..

[29] Smith A., Beales D., O’Sullivan P., Bear N., Straker L. (2017). Low Back Pain with Impact at 17 Years of Age Is Predicted by Early Adolescent Risk Factors From Multiple Domains: Analysis of the Western Australian Pregnancy Cohort (Raine) Study. J. Orthop. Sports Phys. Ther..

[30] Claus A.P., Hides J.A., Moseley G.L., Hodges P.W. (2009). Is ‘ideal’ sitting posture real?: Measurement of spinal curves in four sitting postures. Man. Ther..

[31] Saragiotto B.T., Maher C.G., Yamato T.P., Costa L.O., Costa L.C.M., Ostelo R.W., Macedo L.G. (2016). Motor control exercise for chronic non-specific low-back pain. Cochrane Database Syst. Rev..

[32] McCaskey M.A., Schuster-Amft C., Wirth B., Suica Z., de Bruin E.D. (2014). Effects of proprioceptive exercises on pain and function in chronic neck- and low back pain rehabilitation: A systematic literature review. BMC Musculoskelet. Disord..

[33] Malfliet A., Ickmans K., Huysmans E., Coppieters I., Willaert W., Bogaert W.V., Rheel E., Bilterys T., Wilgen P.V., Nijs J. (2019). Best Evidence Rehabilitation for Chronic Pain Part 3: Low Back Pain. J. Clin. Med..

[34] Georgy E.E. (2011). Lumbar Repositioning Accuracy as a Measure of Proprioception in Patients with Back Dysfunction and Healthy Controls. Asian Spine J..

[35] Lee D., Han S. (2017). Validation of Joint Position Sense of Dorsi-Plantar Flexion of Ankle Measurements Using a Smartphone. Healthc. Inform. Res..

[36] Guidetti L., Placentino U., Baldari C. (2017). Reliability and Criterion Validity of the Smartphone Inclinometer Application to Quantify Cervical Spine Mobility. Clin. Spine Surg..

[37] Ghorbani F., Kamyab M., Azadinia F. (2020). Smartphone Applications as a Suitable Alternative to CROM Device and Inclinometers in Assessing the Cervical Range of Motion in Patients with Nonspecific Neck Pain. J. Chiropr. Med..

[38] Kolber M.J., Pizzini M., Robinson A., Yanez D., Hanney W.J. (2013). The reliability and concurrent validity of measurements used to quantify lumbar spine mobility: An analysis of an iphone^®^ application and gravity based inclinometry. Int. J. Sports Phys. Ther..

[39] Pourahmadi M., Momeni E., Mohseni N., Hesarikia H., Ghanjal A., Shamsoddini A. (2019). The reliability and concurrent validity of a new iPhone^®^ application for measuring active lumbar spine flexion and extension range of motion in patients with low back pain. Physiother. Theory Pract..

[40] Pourahmadi M.R., Taghipour M., Jannati E., Mohseni-Bandpei M.A., Ebrahimi Takamjani I., Rajabzadeh F. (2016). Reliability and validity of an iPhone(^®^) application for the measurement of lumbar spine flexion and extension range of motion. PeerJ.

[41] Bucke J., Spencer S., Fawcett L., Sonvico L., Rushton A., Heneghan N.R. (2017). Validity of the Digital Inclinometer and iPhone When Measuring Thoracic Spine Rotation. J. Athl. Train.

[42] Salamh P.A., Kolber M. (2014). The reliability, minimal detectable change and concurrent validity of a gravity-based bubble inclinometer and iphone application for measuring standing lumbar lordosis. Physiother. Theory Pract..

[43] Banwell H.A., Uden H., Marshall N., Altmann C., Williams C.M. (2019). The iPhone Measure app level function as a measuring device for the weight bearing lunge test in adults: A reliability study. J. Foot Ankle Res..

[44] Balsalobre-Fernández C., Romero-Franco N., Jiménez-Reyes P. (2019). Concurrent validity and reliability of an iPhone app for the measurement of ankle dorsiflexion and inter-limb asymmetries. J. Sports Sci..

[45] Pourahmadi M.R., Ebrahimi Takamjani I., Sarrafzadeh J., Bahramian M., Mohseni-Bandpei M.A., Rajabzadeh F., Taghipour M. (2017). Reliability and concurrent validity of a new iPhone^®^ goniometric application for measuring active wrist range of motion: A cross-sectional study in asymptomatic subjects. J. Anat..

[46] Walter S.D., Eliasziw M., Donner A. (1998). Sample size and optimal designs for reliability studies. Stat. Med..

[47] Lee C.-W., Hyun J., Kim S.G. (2014). Influence of Pilates Mat and Apparatus Exercises on Pain and Balance of Businesswomen with Chronic Low Back Pain. J. Phys. Ther. Sci..

[48] Maher C., Underwood M., Buchbinder R. (2017). Non-specific low back pain. Lancet.

[49] Amundsen P.A., Evans D.W., Rajendran D., Bright P., Bjørkli T., Eldridge S., Buchbinder R., Underwood M., Froud R. (2018). Inclusion and exclusion criteria used in non-specific low back pain trials: A review of randomised controlled trials published between 2006 and 2012. BMC Musculoskelet. Disord..

[50] Dworkin R.H., Turk D.C., Farrar J.T., Haythornthwaite J.A., Jensen M.P., Katz N.P., Kerns R.D., Stucki G., Allen R.R., Bellamy N. (2005). Core outcome measures for chronic pain clinical trials: IMMPACT recommendations. Pain.

[51] Scholz J., Finnerup N.B., Attal N., Aziz Q., Baron R., Bennett M.I., Benoliel R., Cohen M., Cruccu G., Davis K.D. (2019). The IASP classification of chronic pain for ICD-11: Chronic neuropathic pain. Pain.

[52] Koo T.K., Li M.Y. (2016). A Guideline of Selecting and Reporting Intraclass Correlation Coefficients for Reliability Research. J. Chiropr. Med..

[53] Portney L., Watkins M. (2000). Construct Validity. Foundations of Clinical Research: Applications to Practice.

[54] Weir J.P. (2005). Quantifying test-retest reliability using the intraclass correlation coefficient and the SEM. J. Strength Cond. Res..

[55] Gray V.L., Ivanova T.D., Garland S.J. (2014). Reliability of center of pressure measures within and between sessions in individuals post-stroke and healthy controls. Gait Posture.

[56] Bland J.M., Altman D.G. (1986). Statistical methods for assessing agreement between two methods of clinical measurement. Lancet.

[57] Lee A.S., Cholewicki J., Reeves N.P., Zazulak B.T., Mysliwiec L.W. (2010). Comparison of Trunk Proprioception Between Patients With Low Back Pain and Healthy Controls. Arch. Phys. Med. Rehabil..

[58] Wellmon R.H., Gulick D.T., Paterson M.L., Gulick C.N. (2016). Validity and Reliability of 2 Goniometric Mobile Apps: Device, Application, and Examiner Factors. J. Sport Rehabil..

[59] Korakakis V., O’Sullivan K., O’Sullivan P.B., Evagelinou V., Sotiralis Y., Sideris A., Sakellariou K., Karanasios S., Giakas G. (2019). Physiotherapist perceptions of optimal sitting and standing posture. Musculoskelet. Sci. Pract..

